# A plea for more careful scholarship in reviewing evidence: the case of mammographic screening

**DOI:** 10.1259/bjro.20230041

**Published:** 2023-09-25

**Authors:** Stephen W. Duffy, Laszlo Tabar, Tony H.H. Chen, Amy M.F. Yen, Peter B. Dean, Robert A. Smith

**Affiliations:** 1 Centre for Prevention, Detection and Diagnosis, Wolfson Institute of Population Health, Queen Mary University of London, Charterhouse Square, London, UK; 2 Falun Central Hospital, Falun, Sweden; 3 Institute of Epidemiology and Preventive Medicine, College of Public Health, National Taiwan University, Taipei, Taiwan; 4 School of Oral Hygiene, College of Oral Medicine, Taipei Medical University, Taipei, Taiwan; 5 University of Turku, Turku, Finland; 6 American Cancer Society, Atlanta GA, USA

## Abstract

**Objectives:**

To identify issues of principle and practice giving rise to misunderstandings in reviewing evidence, to illustrate these by reference to the Nordic Cochrane Review (NCR) and its interpretation of two trials of mammographic screening, and to draw lessons for future reviewing of published results.

**Methods:**

A narrative review of the publications of the Nordic Cochrane Review of mammographic screening (NCR), the Swedish Two-County Trial (S2C) and the Canadian National Breast Screening Study 1 and 2 (CNBSS-1 and CNBSS-2).

**Results:**

The NCR concluded that the S2C was unreliable, despite the review’s complaints being shown to be mistaken, by direct reference to the original primary publications of the S2C. Repeated concerns were expressed by others about potential subversion of randomisation in CNBSS-1 and CNBSS-2; however, the NCR continued to rely heavily on the results of these trials. Since 2022, however, eyewitness evidence of such subversion has been in the public domain.

**Conclusions:**

An over-reliance on nominal satisfaction of checklists of criteria in systematic reviewing can lead to erroneous conclusions. This occurred in the case of the NCR, which concluded that mammographic screening was ineffective or minimally effective. Broader and more even-handed reviews of the evidence show that screening confers a substantial reduction in breast cancer mortality.

**Advances in knowledge:**

Those carrying out systematic reviews should be aware of the dangers of over-reliance on checklists and guidelines. Readers of systematic reviews should be aware that a systematic review is just another study, with the capability that all studies have of coming to incorrect conclusions. When a review seems to overturn the current position, it is essential to revisit the publications of the primary research.

Systematic reviews are frequently a basis for health policy, and they have informed breast screening policy recommendations.^1–3^ Most major review bodies in public health agree that screening with mammography reduces breast cancer mortality and that the benefits of the intervention outweigh the risks.^1–5^ Areas of disagreement that remain include the appropriate target age range,^1,5^ the quality judgements about the individual trials,^1,3,6,7^ and the dissenting Nordic Cochrane Review (NCR) which concluded that mammographic screening was not justified.^8^ The latter is of particular interest, as despite having had serious flaws pointed out some time ago,^9–11^ and despite the fact that the senior investigator was later expelled from the Cochrane Collaboration,^12^ it is still cited uncritically and clearly continues to be taken seriously by both the research and the policy community.^13,14^

The flaws in the NCR, and its continued citation despite these flaws, raise issues of scholarship in both conducting and interpreting systematic reviews. Systematic reviews are generally considered to be reliable, but they are not infalliable. For example, Mandrik and colleagues in a review of systematic reviews of determinants of participation in breast screening noted that some reviews found no effect of socioeconomic status on screening participation,^15^ despite clear evidence of such an effect from national statistics.^16^

This paper has two aims: to highlight these generally underappreciated issues and to illustrate these with the example of mammographic screening, an important issue in radiology. The issues are most clearly observed in the history of the NCR and its treatment of the evidence from two of the breast screening trials in particular. We summarise these issues, consider the implications for mammography screening, and suggest a way forward to improve the reviewing and interpretation of evidence. This last has implications both for producers and consumers of systematic reviews in all areas of medicine.

## Methods

In this narrative review, we revisited the history of the Nordic Cochrane Review, and its quality assessment of the evidence, specifically on the Swedish Two County Trial (S2C) and the two Canadian National Breast Screening Study (CNBSS-1 and CNBSS-2) trials. We reviewed the major publications of the NCR and the two trials, with the following objectives:

- To identify and illustrate problems in interpretation of primary evidence and of completed systematic reviews;
- To summarise the evolving evidence from the two trials and to identify areas where misinterpretation of the evidence could have been avoided;
- Consider the implications for the effectiveness of mammographic screening;
- Assess the relative importance of primary and secondary research; and
- Develop some recommendations for interpretation of primary and secondary evidence in the future.

## Results

### The Nordic Cochrane Review

The NCR was first published in 2000.^17^ This first review concluded that only the Malmö trial and the NBSS yielded reliable evidence, and that the remaining five trials, the Edinburgh, Gothenburg, S2C, Stockholm, and Health Insurance Plan of Greater New York (HIP) were subject to inadequate randomisation and/or inconsistency. As a result of the exclusion of most the evidence, the results of the NCR’s meta-analysis yielded no significant effect of the offer of mammographic screening on breast cancer mortality. Although this paper focusses on the issues related to the 2CS and the NBSS, it should be noted that at the time, the NCR’s claims with respect to other trials’ quality were contested by independent commentators as well as by ourselves,^18–21^ Also, the NCR methodology was criticised by distinguished trial statisticians Cates and Senn.^22^ Since then, five official updates have been published,^8,23–26^ along with numerous ancillary publications. The most recent publication added the UK Age Trial to the studies which the NCR considered adequately randomised.^8^ The NCR continued to classify the NBSS and Malmö trials as adequately randomised. Strangely, however, the NCR categorised the extension to the latter, Malmö II, which contributed evidence of a mortality benefit of screening in females under age 50, as suboptimally randomised.^8,27^ The conclusions of the official updates have varied in detail, but the substantive conclusion that screening made little or no difference to breast cancer mortality remained.^8^ The review also asserted that mammography screening resulted in very high rates of overdiagnosis of breast cancer.^8^

As noted above, the NCR was at odds with almost every other review by official or quasi-official bodies. The US Preventive Services Task Force, the International Agency for Research on Cancer, the European Commission Initiative on Breast Cancer, the UK Independent Review Group and the American Cancer Society’s reviews all concluded that breast screening with mammography significantly reduced mortality from breast cancer and that the benefits of the intervention outweighed the risks, including those of overdiagnosis.^1–4,28^ The short timeframe and assumptions regarding absolute rates of mortality from breast cancer contribute to the lesser benefits and greater harms estimated by the NCR,^29^ but a major plank in the NCR’s argument was the importance it attached to the CNBSS-1 and 2, and the judgement of low quality which it ascribed to the S2C.^15,25,26^ These judgements of trial quality are still repeated in unqualified terms years later,^6,30,31^ despite the flaws in the judgement having been pointed out in 2004.^11^ It is of value, therefore, to revisit these two trials and re-examine the quality judgements of NCR.

### The Swedish Two-County trial and NCR- design issues

The S2C was initiated in the late 1970s.^32^ In this trial, 45 geographical clusters, within socioeconomically homogeneous strata of sizes 2–3 clusters, were randomised to the offer of mammographic screening (active study population, ASP) or usual care (passive study population, PSP). The screening was by single-view mammography every 33 months in females aged 50–74 and every 24 months in females aged 40–49. After approximately three rounds of screening in the older group and four in the younger group, the PSP was offered a single screen and the trial closed thereafter.^33^

The NCR raised two substantive design issues with the S2C: the possibility of bias due to the cluster randomisation and the ‘exit’ screen of the PSP.^8,17^ When individual randomisation is impractical or may pose a risk of non-adherence to randomisation assignments, cluster randomisation randomises groups (municipalities, health systems, factories, etc.) rather than individuals into invited and control groups. In terms of the cluster randomisation utilised in the S2C, it was noted in 1992 that the average age in the ASP was very slightly greater than that in the PSP, so the design in this case would lead to a bias against invitation to screening if it biased the results at all, because breast cancer risk rises with increasing age.^33^ Since then, statistical analyses of the S2C endpoints have taken into account the cluster randomisation, and results (an approximate 30% reduction in breast cancer mortality with the offer of screening) remained substantially unchanged.^34^ Moreover, a detailed hierarchical Bayesian analysis of the individual cluster data found essentially the same results.^35^ The rigour of this re-analysis was sufficient to convince Dr AB Miller, chief investigator of the Canadian NBSS and an energetic screening sceptic, who stated that he found this analysis ‘particularly compelling in largely dealing with the cluster randomisation issue’.^36^

An exit screen of the control group was built into the S2C to avoid bias in follow-up at the end of the screening rounds due to differential lead time in the two arms. As regards the ‘exit’ screen of the PSP, there are two points which have been made repeatedly,^34^ but which need to be articulated again. First, the 1985 results prior to the completion of this screen show the same 30% reduction in mortality as from later analyses after the exit screen.^32,33^ Secondly, methodological research subsequently showed that the exit screen design was the least biased of a range of screening trial designs.^37^

### The Swedish Two-County trial and NCR- reporting issues

The remaining issues which the NCR had were the fact that the primary endpoint of the S2C was breast cancer mortality, whereas the NCR investigators advocated all-cause mortality, and alleged inconsistencies in reported numbers. As regards the breast cancer mortality endpoint, ascertainment of cause of death was shown to be reliable.^38^ More importantly, we and others have pointed out the strong propensity for all-cause mortality to give misleading results.^39,40^

The claimed ‘inconsistencies’ warrant further exploration at this time. On inspection of the original primary research publications of the Two-County Trial, these can be seen to be spurious. We have carefully reviewed our published results and have found typographical errors in Table 4 of Nixon et al^35^ (1363 erroneously given as 1362), in Table 5 of Tabar et al^41^ (25 incorrectly given as 1025) and in Table 1 of Tabar et al^42^ (22153 incorrectly reported as 22166, 9901 incorrectly reported as 9905 and 75894 incorrectly reported as 75921). These were not, however, the substance of the NCR’s allegations.

First, the NCR investigators objected to the retrospective exclusion of those with prior breast cancer from the randomised population. At the time the S2C was initiated, data linkage was not available to exclude those who already had a prior diagnosis of breast cancer from the study population of more than 130,000 women. These were always excluded from the breast cancer cases and deaths during the trial, as at diagnosis or death, the medical records of the subject were available to the investigators. However, between the 1985 and 1989 publications,^32,42^ linkage with national cancer registration made it possible to exclude these subjects from the denominator. As one would expect, since the numerator (breast cancer death) had already had such cases removed, results with and without such exclusion were almost identical.^32,42^

Other alleged inconsistencies were simply errors on the part of NCR. One example was the difference in total numbers of breast cancer deaths between the Swedish Overview and the S2C publications.^23^ The NCR failed to note that the Swedish overview independently reclassified cause of death and redefined membership of study and control groups for all the Swedish trials, so it would be a cause of suspicion if numbers of deaths reported by the overview were exactly the same as those reported in the original trial publications.^43,44^ More surprisingly, the NCR investigators cited the difference in numbers of deaths between publications reporting at different follow-up times as an ‘inconsistency’.^23^ Specifically, the NCR investigators cited as an inconsistency the fact that in our 1995 publication,^45^ the number of breast cancer deaths in Kopparberg county in hte 40-49 age group was given as 22 in the ASP and 16 in the PSP, whereas in our 1999 paper,^46^ the corresponding figures were 26 and 18.^23^ However, the first of these reported on follow-up to the end of 1992 and the second to the end of 1996. Thus, characterising this increase in numbers of deaths observed with an additional four years of follow-up was an error. One would expect experienced reviewers to understand that a larger number of deaths would have occurred at longer follow-up.^43^

The criticisms of the Two-County Trial were shown to be mistaken at the time of the original NCR publication.^9–11,43^ However, it is difficult for a response to such criticism to achieve the same impact as a study billed as a comprehensive systematic review, and badged with the prestigious name of Cochrane.

### The CNBSS trials and the NCR

The NBSS trials were variously categorised as of ‘medium quality’ and ‘adequately randomised’ by the NCR.^15,47^ The Canadian trials were initiated in 1980 and the first breast cancer mortality results were published in 1992.^48,49^ CNBSS-1 recruited females aged 40–49, and allocated 25,214 to annual mammography plus clinical breast examination, and 25,216 to a single clinical breast examination at baseline, with usual care thereafter. CNBSS-2 recruited females aged 50–59, and allocated 19,711 to annual mammography plus clinical breast examination and 19,694 to annual clinical breast examination only.

Randomisation was by individual, using open lists. Both trials reported no significant or suggestive difference between the mammography and control arms with respect to breast cancer mortality.

Already in the 1990s, there were concerns about possible subversion of the randomisation process due to baseline imbalances in advanced cancers, in particular in CNBSS-1, where there were significantly more advanced cancers at baseline in the mammography arm of the trial.^48^ Bailar and MacMahon conducted a review which found evidence of the lists having been altered, but took the view that the alterations could not have had a substantial effect on results.^50^ However, concerns persisted due to the imbalance previously cited, to the fact that study co-ordinators were aware of the allocation at the time of the initial clinical breast examination, and to the more frequent alterations of names in the lists in the mammography arm of the study.^51^

The NCR dismissed the concerns about subversion of the randomisation, citing the similarity between mammography and control arms with respect to epidemiological risk factors for breast cancer.^17^ This ignores the fact that while shifting of a small number of pre-existing symptomatic cancers from control to mammography group would materially compromise the mortality result, it would have no discernible effect on the proportional distribution of risk factors in the study arms of tens of thousands of females as a whole.^52^ More strangely, NCR uncritically cited the explanation of Miller et al that more than four nodes positive in some cancers in the control group would be more likely to go unrecognised, “…as they were more likely to be treated in centers where careful extensive nodal dissection or evaluation by skilled pathologists was not the norm”.^25,53^ Here, it seems that neither the CNBSS nor the NCR noticed that if the cancers diagnosed in the two groups were treated in centres with differing standards of axillary investigation, then this too would be a source of bias.^43^

Since then, the justifications cited for the imbalance in advanced cancers in the CNBSS-1, while previously unconvincing, have become totally irrelevant, as there is now eyewitness evidence in the public domain that preferential allocation of females with breast lumps to the mammography arm did indeed take place.^54,55^ The concerns about randomisation subversion in the CNBSS-1, first expressed many years ago,^56,57^ have now been shown to be well founded. It has also been remarked that the CNBSS trials were volunteer-based rather than population-based and that the standard of mammography was poor, although the most important issue compromising the validity of the trial is the subversion of the randomisation.^52–57^

## Discussion and recommendations

From the above, it can be seen that the NCR came to unwarranted negative conclusions about the S2C trial on the basis of mistaken interpretation (for example of reports on longer follow-up having larger numbers of deaths) and random detail of subgroup results, while at the same time ignoring clear evidence (which in view of recent revelations can no longer be ignored) of subversion of the randomisation in the CNBSS-1.^34,51–57^ This would be of little importance, except for the fact that the NCR continues to be cited uncritically,^6,13,30,31,58^ perpetuating the myth among clinical colleagues that there is still either equipoise or even a negative effect of breast cancer screening with mammography.

That said, it is generally accepted by authoritative bodies that mammography screening is beneficial and that the benefits outweigh the risks.^2–5^ The publications of NCR have only caused unnecessary controversy and confusion, which could have been avoided had the warnings about its quality of evidence judgements been heeded in the past,^9–11^ and if the Cochrane Collaboration had taken steps to withdraw the NCR report. This is a question wider than the reliability or otherwise of CNBSS-1, CNBSS-2 and NCR. The important question is how can we reduce the risks of such confusion in future? A number of suggestions may be made.

1. There needs to be greater awareness that the gold standard of evidence is not systematic review or meta-analysis. The gold standard is high-quality primary research.
2. It is generally accepted that all studies are capable of returning the wrong answer. What is less appreciated is that a systematic review is just another study, and so is similarly capable of coming to incorrect conclusions.
3. The implication of the above for those reading or referring to systematic reviews is that a systematic review cannot be assumed to be infallible. If a systematic review claims to find that the current consensus is wrong, due to the quality of evidence of the primary research, it is important for scientific and policy colleagues to revisit the primary research to ascertain whether the review’s conclusion stands up.
4. For those carrying out systematic reviews, there is clearly a need to rely more on scholarship and thought and less on checklists and box-ticking. When a study is potentially informative, the question should not be whether it ticks this box or that, but what it tells us. One would prefer to use individual randomisation in a trial, but that does not mean that cluster-randomised trials tell us nothing. On the other hand, an individually randomised trial may be compromised by failure to follow the randomisation protocol.
5. Lesser pursuit of exclusion would be valuable. When Sir Richard Peto and colleagues first produced their ground-breaking meta-analyses, the motivation was to bring all the trial evidence together because the individual trials were sometimes too small to speak for themselves.^59^ Nowadays, the emphasis in systematic reviewing is to amass a very large number of studies using broad search criteria, and subsequently to exclude the vast majority of these. The problem with this approach is that for any study, it is easy to find one or other criterion which the study could be argued to fail to satisfy. Indeed, it is not unusual for a systematic review to find no relevant studies.^60^
6. Arguably, a more productive approach, particularly if the health question is a very important one, is to draw together all the relevant evidence, including non-trial evidence if it is informative, as in the International Agency for Research on Cancer Handbooks.^61^

The force of the points above can be more clearly seen if one considers a hypothetical example from another area of medicine. If one read a systematic review which concluded that statins reduce cholesterol, it would be reasonable to accept it at face value. If, on the other hand, a systematic review concluded that the received wisdom was wrong and that statins did not reduce cholesterol, on the basis that the trials which found such a reduction were of low quality, what is the first thing that a reader would do? One would revisit the original primary research to assess whether the review’s judgement of trial quality is correct. If policymakers and research colleagues had done this following the initial publication of the NCR, much subsequent confusion could have been avoided.

In conclusion, a more thoughtful approach to drawing together published evidence is indicated. While the well-developed technology of systematic reviewing with its stepped procedures and use of checklists is economic of researcher time, it can get the wrong answer as the NCR did. Early detection of breast cancer with mammography screening, and treatment at an early stage of disease, reduces mortality from breast cancer, and only a highly skewed interpretation of the trial data can suggest otherwise. More importantly, the consumers of systematic reviews need to be on their guard against erroneous conclusions, particularly when a review declares that the current position on a particular intervention is wrong. In such a case, there is no substitute for careful study of the original primary research.
